# Morphine, Oxygen, Nitrates, and Mortality Reducing Pharmacological Treatment for Acute Coronary Syndrome: An Evidence-based Review

**DOI:** 10.7759/cureus.2114

**Published:** 2018-01-25

**Authors:** José Nunes de Alencar Neto

**Affiliations:** 1 Departamento De Cardiologia, Hospital São Paulo

**Keywords:** cardiology, adjuvant treatment, myocardial infarction, acute coronary syndrome, morphine, aspirin, oxygen, nitrate

## Abstract

Since it was first reported in 1912, acute coronary syndrome (ACS) has become the leading cause of death in the Western world. Several improvements that have been made over the years in the pharmacological treatment of ACS have reduced the relative risk of death due to myocardial infarction from 35-45% previously to approximately 3.5% at present. Universities, websites, and educational videos commonly use a mnemonic for morphine, oxygen, nitrates, and aspirin (MONA) to refer to the adjuvant treatment used for the management of ACS.

We review the scientific data pertaining to treatment strategies for the management of ACS and discuss whether MONA remains relevant in the present scenario.

While using morphine and oxygen is associated with risks such as higher mortality and increase in the size of the infarct, respectively, several available drugs such as fibrinolytics, anticoagulants, beta-blockers, renin-angiotensin-aldosterone system inhibitors, P2Y12 inhibitors, and statins are known to be useful to treat ACS.

MONA should be viewed as an obsolete teaching and learning aid, and therefore we recommend that its use be discontinued for the management of ACS.

## Introduction and background

Acute coronary syndrome (ACS) is the leading cause of death in the Western world. In the United States, approximately one in seven deaths is secondary to coronary heart disease. It is estimated that more than one million Americans experience a myocardial infarction (MI) every year, which in effect means that one American experiences an MI every 24 seconds [1].

Diagnostic and treatment modalities have vastly evolved over the years. Since this disease entity was first reported by Herrick in 1912 [2], adjuvant and pharmacological modalities for the management of ACS have evolved greatly and resulted in better pain relief and mortality reduction after cardiac events. Presently, use of a few drugs such as aspirin, heparin, or P2Y12 inhibitors is mandatory to treat ACS.

Unfortunately, the mnemonic MONA (morphine, oxygen, nitrates, and aspirin) continues to be used as a teaching tool in universities, on websites, and in educational videos. It must be stated here that a few less experienced professionals who learn this mnemonic might in fact be doing harm to their patients because strict adherence to only MONA may cause underestimation of the importance of other drugs not contained in MONA.

In this article, we discuss historical and technical aspects of the pharmacological treatment used in the management of ACS and the use of this mnemonic and investigate whether MONA continues to be relevant in the contemporary management of ACS.

## Review

Evidence-based aspects regarding morphine, oxygen, aspirin, and nitrates (MONA)

*Morphine*     

Authors of the 'Can Rapid Risk Stratification of Unstable Angina Patients Suppress Adverse Outcomes with Early Implementation of the ACC/AHA Guidelines (CRUSADE) Registry' [3] evaluated outcomes in patients who presented with an non-ST-elevation myocardial infarction (NSTEMI) and were administered morphine within 24 hours of presentation. This being an observational study, patients were not randomized. Evaluation of more than 57,000 patients revealed that the adjusted odds ratio of death rose from 4.7% to 5.5% (adjusted odds ratio (OR) 1.48, 95% confidence interval (CI) 1.33-1.64). The adjusted odds-ratio (OR) for myocardial infarction (adjusted OR 1.34, 95% CI 1.22-1.48) was superior among patients who received morphine. Based on adjusted subgroup analysis, morphine showed a significantly poor performance among all parameters used. The hypotheses that explain these findings are: (1) patients not treated with morphine did not receive optimal medical treatment. However, this is not true based on the registry because those who received medication were more likely to have received aspirin, heparin, clopidogrel, diagnostic coronary angiography, and percutaneous coronary intervention (PCI), (2) morphine use may be an indicator of a seriously ill patient because it is usually prescribed for patients with acute pulmonary edema or refractory pain. However, this presumption was also determined to be untrue in the study, (3) analgesic effects may mask the severity of angina and result in misinterpretation of signs and symptoms. This presumption was proved to be untrue for the same reason presented in (1). A possible reason to explain this harmful effect is that morphine is associated with delayed activity of platelet inhibitor drugs in patients presenting with ST-elevation myocardial infarction (STEMI) [4].

Owing to these negative findings, morphine has been downgraded a class IIb indication with level of evidence (LOE) B based on American Heart Association/American College of Cardiology (AHA/ACC) guidelines [5].

Regarding STEMI, to date, the best evidence is based on the 'Administration of Ticagrelor in the cath Lab' or in the 'Ambulance for New ST Elevation Myocardial Infarction to Open the Coronary Artery' (ATLANTIC) trial [6]. This study compared pre- versus in-hospital treatment using ticagrelor (a P2Y12 inhibitor) with respect to electrocardiographic and angiographic outcomes. Subgroup analysis showed that patients who did not receive morphine demonstrated a significant improvement in the electrocardiogram (ECG)-based endpoint (defined as an improvement in > 70% of the ECG obtained prior to performing the PCI). Despite growing negative evidence associated with its use in patients diagnosed with STEMI, the AHA/ACC guidelines continue to classify morphine as the drug of choice for pain relief in patients with STEMI [7].

Oxygen

One of the most robust data pertaining to the use of oxygen is provided by the 'Air Versus Oxygen in Myocardial Infarction' (AVOID) trial [8]. This was a multicenter, randomized-controlled trial that compared the effects of the administration of oxygen (8 L/min) versus no supplemental oxygen in patients with STEMI and oxygen saturation ≥ 94% measured using a pulse oximeter. The primary endpoint was infarct size measured using troponin and cardiac enzyme assays. Secondary endpoints included recurrent myocardial infarction (MI) and cardiac arrhythmias. At six-month follow-up the secondary endpoints were observed to be statistically significant favoring a no-oxygen strategy: in-hospital recurrent MI (5.5% x 0.9%, P = 0.006) and major cardiac arrhythmias (40.4% x 31.4%, P = 0.05). In 2017, the 'Determination of the Role of Oxygen in Suspected Acute Myocardial Infarction' (DETO2X-AMI) trial enrolled acute coronary syndrome (ACS) patients with an oxygen saturation of ≥ 90% to receive oxygen supplementation versus inhalation of ambient air. There was no statistically significant difference in one-year all-cause mortality observed between groups [9].

NSTEMI and STEMI AHA/ACC guidelines recommend the use of supplemental oxygen when oxygen saturation is ≤ 90% [5,7].

Nitrates

There is lack of sufficient evidence regarding its use. Randomized-controlled trials have shown that its systemic use, not directed to symptoms, could not provide benefit in terms of improved mortality or adverse cardiovascular outcomes [10,11].

NSTEMI and STEMI AHA/ACC guidelines recommend administration of sublingual or intravenous nitrates for management of angina, hypertension, acute pulmonary edema, or recurrent ischemia as a class I indication [5,7].

Aspirin

In 1988, the 'Second International Study of Infarct Survival' (ISIS-2) established the beneficial effect of aspirin when administered as a 160 mg-dose during the acute phase of MI. With more than 17,000 patients enrolled, the group receiving aspirin showed a 23% reduction in cardiovascular mortality in five weeks. (9.4% x 11.8%, P < 0.00001) [12].

Aspirin is a vital component of the optimal treatment regimen employed for the management of ACS. It is classified as having a class I indication, LOE A or B based on the primary NSTEMI [5] and STEMI [7] AHA/ACC guidelines.

Past, present, and future of acute coronary syndrome treatment

Since MI was first described by Herrick in 1912 [2], physicians and scientists globally continued their efforts to gain a better understanding of this disease entity that soon went on to become a major cause of mortality in developed countries. In earlier times, initiation of the treatment plan would begin with two to three weeks of bed rest at a place far from the nurses’ station so that the patient would not be emotionally stressed. Braunwald wrote, “It was not uncommon to arrive on the medical floor at 6 a.m. to discover that one of the patients had died quietly during that night” and that “older physicians accepted it ‘just the way it was’” [13].

In the 1950s 'Harrison’s Principles of Internal Medicine' outlined the treatment of MI as being limited to the administration of oxygen, morphine, and nitroglycerin [14]. In the 60s, Desmond Julian established the first Coronary Unit [15], an area where only patients with MI would be admitted and would receive continuous electrocardiographic monitoring performed by medical and nursing staff trained to perform careful chest resuscitation. The advent of coronary units led to a reduction in the in-hospital mortality after ACS from 29% in the 60s to 15% at the beginning of the era of reperfusion and mortality reduction [16].

MONA was the standard treatment regimen used for ACS in the late 80s. However, it did not include fibrinolytics—a very important class of drugs, which had already been tested at the time. Subsequently, several other drugs have been discovered and tested, as we will review in the next section (Table 1).

**Table 1:** Mortality trends secondary to acute coronary syndrome over 100 years The first era was marked by treatment directed toward pain relief and bed rest. The second era began in the early 60s with the development of the first coronary unit, leading to a 50% relative mortality reduction. The third era was marked by the search for reperfusion agents represented by fibrinolytics and also for other drugs that showed reduction in mortality and in major cardiac adverse events (primarily the discovery of aspirin and anticoagulants). This era extended into the new century when P2Y12 inhibitors were developed and statins became an integral component of the acute treatment regimen for management of acute coronary syndrome. Abbreviations: RAAS = Renin-angiotensin-aldosterone system

Era	Agent/Strategy	In-hospital mortality
1912-1961 (bed rest and expectant treatment)	Bed Rest Morphine Oxygen Nitrates	35-45%
1960-1986 (the coronary care unit)	Coronary Units	15-20%
1986–2000 (myocardial reperfusion)	Fibrinolytics Aspirin Anticoagulants Beta-blockers RAAS inhibitors	7%
2000-ahead	P2Y12 inhibitors Statins Angioplasty with stents	3.5%
90% relative risk reduction over the past 100 years		

A summary of the recommendations based on AHA/ACC guidelines is shown in Table 2.

**Table 2:** Drugs and strategies currently available for acute coronary syndrome treatment Abbreviations: AHA = American Heart Association. ACC = American College of Cardiology. ACE = angiotensin-converting enzyme. ARB = angiotensin receptor blockers. NSTEMI = non-ST elevation myocardial infarction. PCI = percutaneous coronary intervention. RAAS = renin angiotensin-aldosterone system. STEMI = ST-elevation myocardial infarction.

Agent	Comments	AHA/ACC Recommendations
Morphine	No mortality benefit. Associated with higher risk of death in patients presenting with NSTEMI and perhaps a higher infarct size in patients presenting with STEMI.	NSTEMI: Class IIb.
		STEMI: considered the drug of choice for pain relief, but without a specific recommendation level.
Oxygen	No mortality benefit. Associated with a higher risk of recurrent MI and major cardiac arrhythmias.	NSTEMI: Class I in when oxygen saturation ≤ 90%.
		STEMI: indicated when oxygen saturation ≤ 90% without a specific recommendation level.
Nitrates	No mortality benefit.	NSTEMI: Class I in a setting of persistent ischemia, heart failure or hypertension.
		STEMI: in a setting of persistent ischemia, heart failure or hypertension without a specific recommendation level in STEMI guidelines.
Aspirin	23% mortality reduction.	Class I in NSTEMI and STEMI guidelines.
Fibrinolytics	18% mortality reduction.	NSTEMI: Class III.
		STEMI: Class I in for duration of symptoms < 12 hours and within 30 minutes of hospital arrival.
PCI	Preferred over fibrinolytics after a meta-analysis showed a reduction in death favoring PCI.	Class I in NSTEMI and STEMI guidelines.
Anticoagulants	Reduction in mortality and major cardiac adverse events	Class I in NSTEMI.
		STEMI with planned primary PCI: unfractionated heparin and bivalirudin receive class I; STEMI with fibrinolytic therapy: unfractionated heparin, enoxaparin and fondaparinux receive Class I.
Beta-blockers	Unclear benefit. Effective in relieving pain and ischemia.	Class I in NSTEMI and STEMI guidelines.
RAAS inhibitors	Mortality reduction. Although ARBs receive class I of indication in the setting of ACE inhibitors intolerance, one should keep in mind that Losartan led to a non-significantly higher mortality compared to captopril in ACS patients.	NSTEMI: Class I for patients with ejection fraction ≤ 40%, ARB for those intolerant to ACE inhibitors and Class IIa for all patients.
		STEMI: Class I for patients with ejection fraction ≤ 40%, ARB for those intolerant to ACE inhibitors and Class IIb for all patients
P2Y12 inhibitors	Reduction in major cardiac adverse events.	NSTEMI: clopidogrel and ticagrelor are class I.
		STEMI: clopidogrel, prasugrel and ticagrelor are class I when primary PCI is performed and only clopidogrel is Class I to support fibrinolytic therapy.
Glycoproteins IIb/IIIa inhibitors	No mortality benefit.	NSTEMI: is Class I in high-risk patients not adequately pre-treated using P2Y12 inhibitors.
		STEMI: Class IIa in conjunction with unfractionated heparin when primary PCI is performed.
Statins	Reduction of recurrent ischemia.	Class I in NSTEMI and STEMI guidelines.

Historical and scientific aspects of reperfusion and mortality reduction era   

Fibrinolytics

Growing evidence of the benefit of fibrinolytic therapy in the early 80s motivated the publication of the report of the 'Gruppo Italiano per lo Studio della Sopravvivenza nell’Ínfarto miocardico' (GISSI-1) trial in 1986 [17]. It was an important trial involving 11,806 patients enrolled in 176 coronary units. There was an 18% reduction in mortality during follow-up (10.7% x 13%, P = 0.0002) in patients using streptokinase (SK) compared to those who used standard therapy, which at that time was primarily based on providing symptomatic relief. The authors also noticed that the earlier SK was infused, the greater the extent of benefit. This marked the beginning of a new era in reperfusion therapy.

In 1993, the 'Global Utilization of Streptokinase and Tissue Plasminogen Activator for Occluded Coronary Arteries' (GUSTO-1) trial randomly assigned more than 41,000 patients diagnosed with STEMI to receive either alteplase (t-PA) or SK. Administration of subcutaneous versus intravenous heparin was also tested, and aspirin as well as beta blockers were administered as additional therapy. Therapy with t-PA and intravenous heparin showed a statistically significant 14% reduction in 30-day mortality [18]. This marked the beginning of the era where cardiologists coined the phrase ‘time is muscle’ to indicate that loss of time in initiating treatment was directly proportional to the loss of heart muscle.

Fibrinolysis is not indicated in patients with NSTEMI [5]. In those presenting with STEMI, fibrinolytics are indicated as class I, LOE A in patients complaining of pain of < 12 hours duration, and its infusion should be started within 30 minutes of hospital arrival, although presently, primary PCI is preferred [7], and this strategy has shown better outcomes—a reduction in short-term mortality (7% x 9%, P = 0.0002), non-fatal re-infarction (3% x 7%, P = 0.0001), and stroke (1% x 2%, P = 0.0004) [19].

Anticoagulants

It was only in the 90s that large trials began to be performed to clarify this issue. The GISSI-2 trial enrolled 12,381 patients and randomly assigned them to receive SK or alteplase and heparin (12,500 U twice daily beginning at 12 hours after fibrinolytic administration) therapy with aspirin and a beta blocker. Heparin could not lead to a reduction in the combined end-point of death and severe left ventricular damage (22.7% x 22.9%, CI 0.91-1.08) but led to a higher risk of bleeding [20].

In 1992, a larger randomized-controlled trial was published called the 'International Study of Infarct Survival' (ISIS-3), which randomized 41,299 patients to test the effect of SK, duteplase, or anistreplase and also aspirin alone versus aspirin added to 12,500 U of heparin administered four hours after infusion of a fibrinolytic. This trial also did not show a significant association between the use of these agents with reduced mortality (7.4% x 7.9%, P = 0.06). However, combining the results of the GISSI-2 and ISIS-3 trials, a significant reduction in mortality was observed (6.8% x 7.3%, P < 0.01) [21].

The role of enoxaparin, a low-molecular-weight substance which inhibits Factor Xa of the coagulation cascade, was compared in a setting of NSTEMI and STEMI in the final decade of the last century and the first decade of the new millennium. The 'Superior Yield of the New strategy of Enoxaparin, Revascularization, and GlYcoprotein Inhibitors' (SYNERGY) trial published in 2004 showed that enoxaparin was non-inferior to unfractionated heparin in patients with NSTEMI [22]. The 'Enoxaparin and Thrombolysis Reperfusion for Acute Myocardial Infarction Treatment Thrombolysis in Myocardial Infarction' (ExTRACT-TIMI 25) trial performed in 2006 was a randomized controlled trial that involved 20,506 patients who received intravenous unfractionated heparin with maintenance of an activated partial-thromboplastin time at 1.5 to 2.0 times the control value or subcutaneous enoxaparin based on age and renal function. The primary end-point was a composite of death from any cause of non-fatal MI in the first 30 days. The primary outcome was statistically significantly reached (9.9% x 12.0%, P < 0.001) in 30 days, in addition to the outcome that tested safety against major bleeding (2.1% x 1.4%, P < 0.001) [23].

The role of fondaparinux, a selective inhibitor of Factor Xa was also tested in a setting of NSTEMI and STEMI. The 'Organization to Assess Strategies for Ischemic Syndromes' (OASIS-5) trial studied 20,078 patients with NSTEMI and showed a similarity between fondaparinux and enoxaparin in reducing the risk of ischemic events over a follow-up of six months, but they were observed to show a significant reduction in major bleeding and the risk of death [24]. OASIS-6 blindly randomized 12,092 patients with STEMI to receive fondaparinux versus placebo or unfractionated heparin. There was a reduction in deaths observed in the fondaparinux group, except in those who underwent PCI, who showed an increased predisposition to catheter thrombosis [25].

Bivalirudin is a direct thrombin inhibitor. The 'Harmonizing Outcomes with Revascularization and Stents in Acute Myocardial Infarction' (HORIZONS-AMI) trial compared bivalirudin to unfractionated heparin in patients with STEMI undergoing primary PCI [26]. Results showed that use of bivalirudin alone led to significantly lower 30-day rates of death from cardiac causes (1.8% vs. 2.9%, P = 0.03) and all-causes (2.1% vs. 3.1%, P = 0.047) compared to the use of unfractionated heparin plus glycoprotein IIb/IIIa inhibitors. Later, the 'How Effective are Antithrombotic Therapies in Primary Percutaneous Coronary Intervention' (HEAT-PPCI) trial randomized 1917 patients at a single center to analyze the composite outcome of all-cause mortality, stroke, re-infarction, or unplanned revascularization. The composite outcome was more frequently observed in the bivalirudin group (8.7% x 5.7%, P = 0.01) [27]. Results of a multicenter randomized, open-label trial involving NSTEMI and STEMI patients with planned PCI published in 2017 showed no difference between heparin and bivalirudin in a composite outcome of death from any cause, MI, and major bleeding in 6006 patients [28].

The most recent AHA/ACC guidelines on STEMI indicate a class I routine prescription for unfractionated heparin, with or without glycoprotein IIb/IIIa inhibitors, in planned primary PCI. Bivalirudin is class I. Fondaparinux is class III and, therefore, is contraindicated in this setting. When fibrinolytic therapy is planned, unfractionated heparin, enoxaparin and fondaparinux are indicated as class I [7]. In those with NSTEMI, all drugs have a class I indication [5].

Beta Blockers

Between 1974 and 1984, several small trials have reported a lower mortality rate in studied patients when a beta-blocker was prescribed within the first one to three weeks after an MI. It was the first drug ever to show mortality reduction in ACS [29]. It should be prescribed within the first 24 hours of the cardiac event and is associated with a lower incidence of re-infarction and recurrent chest pain [30].

In NSTEMI and STEMI ACC/AHA guidelines, it has a class I, LOE A or B indication [5,7].

Renin-Angiotensin-Aldosterone System (RAAS) Inhibitors

For several years, it had been believed that hypotension would decrease coronary blood flow, and thus, would be harmful in ACS patients. However, the 'Sleep Apnea Cardiovascular Endpoints' (SAVE) trial showed that in post-myocardial infarction patients with an ejection fraction ≤ 40%, captopril was associated with a 19% reduction in mortality (20% x 25%, P = 0,019) [31]. In 1996, the 'Gruppo Italiano per lo Studio della Sopravvivenza nell’Ínfarto miocardico' (GISSI-3), a huge randomized controlled trial involving 43,047 MI patients showed that use of lisinopril was associated with an 11% mortality reduction compared to a placebo (6.3% x 7.1%, P = 0.03) in a six-week follow-up [10].

Because angiotensin II is the prototype of RAAS, it would be logical to block this system by antagonizing AII at the level of its receptor. This therefore led to the development of the direct angiotensin receptor blockers (ARB) [32].

The 'Valsartan In Acute Myocardial Infarction' (VALIANT) trial compared valsartan with captopril in a setting of acute MI and showed it to be non-inferior to the angiotensin-converting enzyme inhibitor (ACE-I) after an acute MI with heart failure or left ventricular dysfunction [33]. The 'Optimal Trial in Myocardial Infarction with the Angiotensin II Antagonist Losartan' (OPTIMAAL), a randomized controlled trial involving 5477 patients compared captopril with losartan in high-risk post-MI patients and a non-significant higher mortality rate was observed in the group administered ARBs leading to the conclusion that this drug is not suitable for use in this patient population [34].

In cases of NSTEMI, high-risk patients and those with an ejection fraction ≤ 40% are categorized as having a class I recommendation for ACE-I. ARBs are also categorized as class I, but only in the setting of intolerance to ACE-I. Patients without systolic dysfunction have a IIb recommendation for using this drug [5]. In cases of STEMI, patients with an ejection fraction ≤ 40% are categorized as having a class I recommendation for ACE-I and ARBs for those intolerant to the former, and a class IIa recommendation in the absence of systolic dysfunction [7].

P2Y12 Inhibitors

The 'Clopidogrel in Unstable Angina to Prevent Recurrent Events' (CURE) trial was the first to test the efficacy of a combination of aspirin and clopidogrel in the setting of ACS—what is commonly called dual antiplatelet therapy (DAPT). In this study, 12,562 NSTEMI patients were randomly assigned to study groups, and the composite outcome of death from cardiovascular causes, nonfatal MI or stroke was observed to be reduced in 20% (9.3% x 11.4% P < 0.001) although there was noted to be increased major (but not life-threatening) bleeding [35]. The 'Clopidogrel as Adjunctive Reperfusion Therapy-Thrombolysis in Myocardial Infarction 28' (CLARITY-TIMI-28) trial studied 3491 randomly assigned patients who were candidates for fibrinolytic treatment. In this study, a loading dose of clopidogrel of 300 mg (four pills) followed by a 75 mg daily dose was tested against a placebo. The study results published in 2006 showed a reduction of 36% in composite endpoint odds (15% x 21.7%, P < 0.001) without increasing bleeding rates [36].

Prasugrel is a third-generation thienopyridine that inhibits the P2Y12-ADP receptor with a more rapid rate of action and more potent platelet inhibition than clopidogrel [37]. The 'Trial to Assess Improvement in Therapeutic Outcomes by Optimizing Platelet Inhibition with Prasugrel–Thrombolysis in Myocardial Infarction' (TRITON-TIMI 38) compared prasugrel with clopidogrel in 13,608 STEMI and NSTEMI patients who were candidates for a planned PCI. A 19% reduction in composite endpoint was observed with prasugrel (9.9% x 12.1%, P < 0.001); however, a significant increase in major bleeding, including fatal blood loss was observed. The caveats from this trial are: 1) clinicians should be mindful of the increased risk of major bleeding associated with this drug and must therefore exercise caution before prescribing it, 2) a post-hoc analysis demonstrated that patients aged ≥ 75 years or with a body weight ≤ 60 kg are not benefitted and patients with previous stroke may in fact experience potential adverse effects, 3) this drug should be administered in ACS patients who have been treated with PCI [38]. The 'Targeted Platelet Inhibition to Clarify the Optimal Strategy to Medically Manage Acute Coronary Syndromes' (TRILOGY ACS trial) studied the effect of prasugrel compared to clopidogrel in patients who do not undergo revascularization and found no significant differences between them [39].

Ticagrelor is a cyclopentyl triazolopyrimidine with a faster onset and offset of action and shows more potent platelet inhibition than clopidogrel [40]. The 'Platelet Inhibition and Patient Outcomes' (PLATO) trial compared ticagrelor to clopidogrel in 18,624 ACS patients with and without ST elevation. There was a 16% reduction observed in the composite endpoint favoring ticagrelor (9.8% x 11.7%, P < 0.001) [41].

Cangrelor is a novel nonthienopyridine adenosine triphosphate analogue. It is a compound administered intravenously that produces potent, rapid-acting, and reversible P2Y12 inhibition—onset of action observed within two minutes, maximum inhibition in 30 minutes, and function restoration after 60 minutes of cessation of administration of the drug [42]. When directly compared to clopidogrel, cangrelor was noted to reduce stent thrombosis and MI [43].

In NSTEMI guidelines, clopidogrel and ticagrelor have a class I, LOE B indication for at least 12 months after ACS in patients treated with stents [5]. In patients with STEMI, clopidogrel, ticagrelor, and prasugrel are all categorized as having a class I indication when primary PCI is performed and only clopidogrel is class I to support fibrinolytic therapy [7].

Statins

Statins early use was tested in a setting of ACS in controlled trials by A to Z, that compared early initiation of intensive therapy (simvastatin at 40 mg/day in the first month, later increased to 80 mg/day) versus administration of placebo for four months and then simvastatin at 20 mg/day.The study showed a statistically significant reduction in recurrent angina, MI, new-onset ACS, stroke, or cardiovascular death (OR 0.75, P = 0.02) in the intensive therapy group, although these patients showed a higher incidence of myopathy [44].

Later, the 'Myocardial Ischemia Reduction with Aggressive Cholesterol Lowering' (MIRACL) trial randomized 3086 patients diagnosed with ACS without ST-segment elevation for treatment with 80 mg/d of atorvastatin or placebo initiated within 24-96 h after the cardiac event in a 16-week follow-up study. The primary composite outcome defined as all-cause mortality, fatal and non-fatal acute MI, cardiac arrest, and recurrent ischemia was reduced from 17.4% in the placebo group to 14.8% in the atorvastatin group (OR 0.84, P = 0.048) with results showing a significant reduction in recurrent ischemia, but not the other endpoints [45].

The 'Pravastatin or Atorvastatin Evaluation and Infection Therapy–Thrombolysis in Myocardial Infarction 22' (PROVE IT-TIMI 22) was another randomized controlled trial with the same objectives and evaluated the efficacy of treatment with atorvastatin at a dose of 80 mg/d versus moderate intensity treatment with pravastatin at a dose of 40 mg/d at least 10 days after the cardiac event. Results showed superior efficacy of the intensive regimen in reducing the primary endpoint of all-cause death, MI or unstable angina and revascularization [46].

The 'Improved Reduction of Outcomes: Vytorin Efficacy International Trial' (IMPROVE-IT) trial compared the efficacy of simvastatin (40 mg/d) with or without ezetimibe (10 mg/d) at least 10 days after the cardiac event. Results showed an improvement in cardiovascular outcomes defined as cardiovascular death, nonfatal MI, unstable angina or stroke, and revascularization (hazard ratio (HR) 0.936, P = 0.016) after a seven-year follow-up [47].

High-intensity statins are categorized as having a class I indication, and statin therapy should be initiated as soon as possible unless contraindicated based on ACC/AHA NSTEMI [5] and STEMI [7] guidelines.

In-hospital mortality decreased from 29% in 1969 [16] to approximately 5-7% presently [48,49] and calculated as 3.5% in patients enrolled in clinical trials. Between 2003 and 2013 the annual death rate attributable to coronary heart disease in the US declined from 38% to 22.9% [1].

## Conclusions

There has been a dramatic improvement in the pharmacological treatment of ACS patients over the last six decades associated with a 90% decline in the mortality trends of this syndrome. We reviewed the role of drugs used for the management of ACS represented by the MONA mnemonic. To summarize, morphine is of limited value because its use is shown to be associated with increased mortality in patients with NSTEMI; oxygen therapy needs to be administered with caution and should be administered preferably in patients with an oxygen saturation ≤ 90%, because it may precipitate recurrent MI; nitrates show excellent analgesic effect but must not be used systemically; and aspirin must be used in all patients except in those with contraindications to its use, because robust data has demonstrated a 23% reduction in cardiovascular mortality associated with the use of this drug.

Following the GISSI-1 trial, which indicated the potent beneficial effects of fibrinolytics, and the ISIS-2 trial, which investigated the role of aspirin and proved its efficacy, ACS treatment has undergone a dramatic transformation with the development and use of a greater number of improved drugs that have effectively contributed to mortality reduction and better outcomes. We reviewed the important role of anticoagulants, P2Y12 inhibitors, fibrinolytics, beta-blockers, RAAS inhibitors, and statins.

It would be appropriate to conclude that the MONA mnemonic should be viewed as an obsolete teaching and learning aid. Continuing to use the outdated MONA mnemonic may negatively affect the treatment of ACS patients because other drugs that effectively reduce mortality or coronary outcomes are not contained in this mnemonic. A newly trained student or a less experienced physician may misinterpret MONA as being sufficient to treat ACS. Therefore, we recommend its use be discontinued for the management of ACS.
